# Supplementary material for: Solid-Phase “Self-Hydrolysis” of [Zn(NH3)4MoO4@2H2O] Involving Enclathrated Water—An Easy Route to a Layered Basic Ammonium Zinc Molybdate Coordination Polymer
Source: Molecules. 2021 Jun 30;26(13):4022. doi: 10.3390/molecules26134022 (PMC8272139; doi:10.3390/molecules26134022)
Supplement: Supplementary file 1 [file molecules-26-04022-s001.zip › molecules-1263235-supplementary.pdf]

ESI

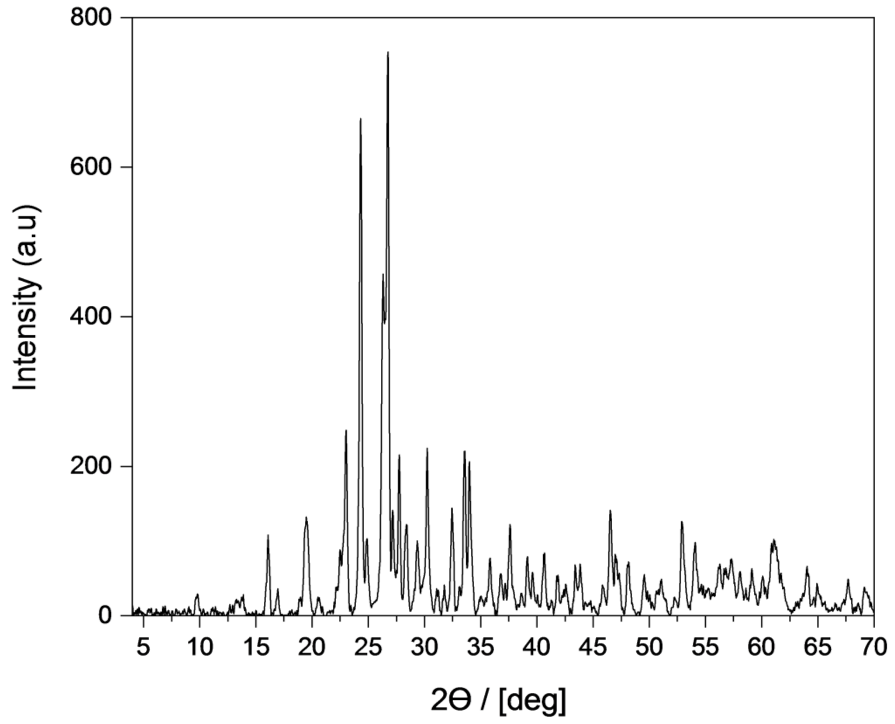

Figure S1. Thermal decomposition product of **1**@2H_2O under air ($ZnMoO_4$)

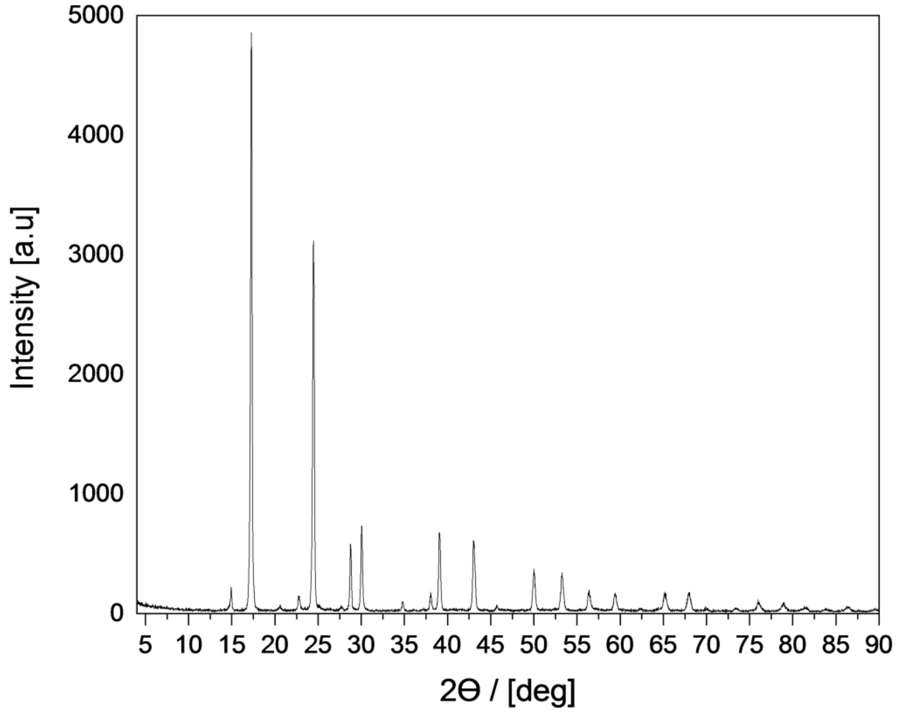

5000
4000
3000
2000
1000
0
Intensity [a.u]
5 10 15 20 25 30 35 40 45 50 55 60 65 70 75 80 85 90
2Θ / [deg]

a)

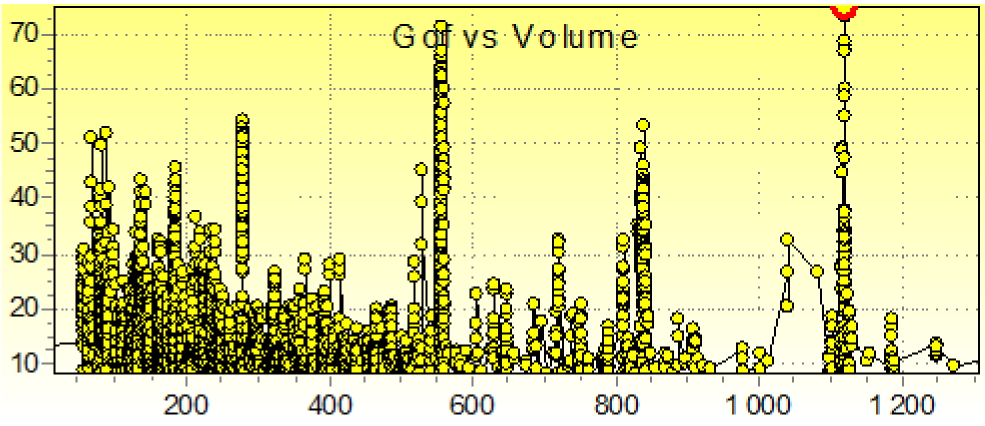

Gof vs Volume
70
60
50
40
30
20
10
200 400 600 800 1 000 1 200

(b)

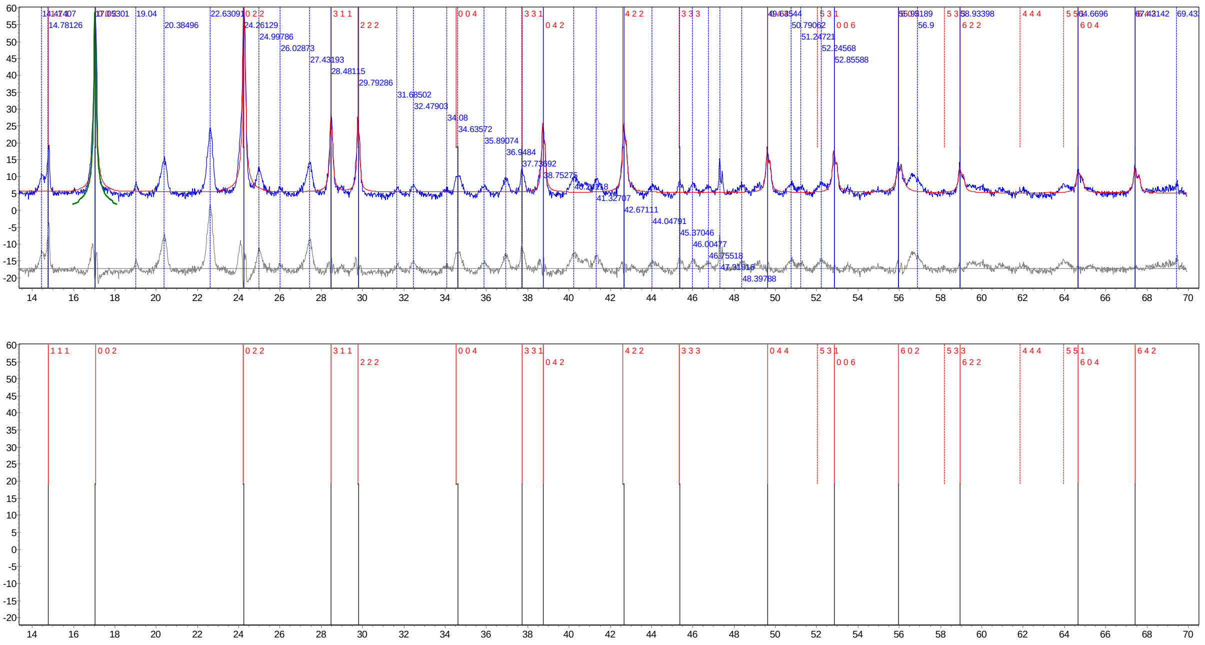

(c)

Figure S2. XRD (a) and indexing results (b, c) of compound **1**@$2H_2O$

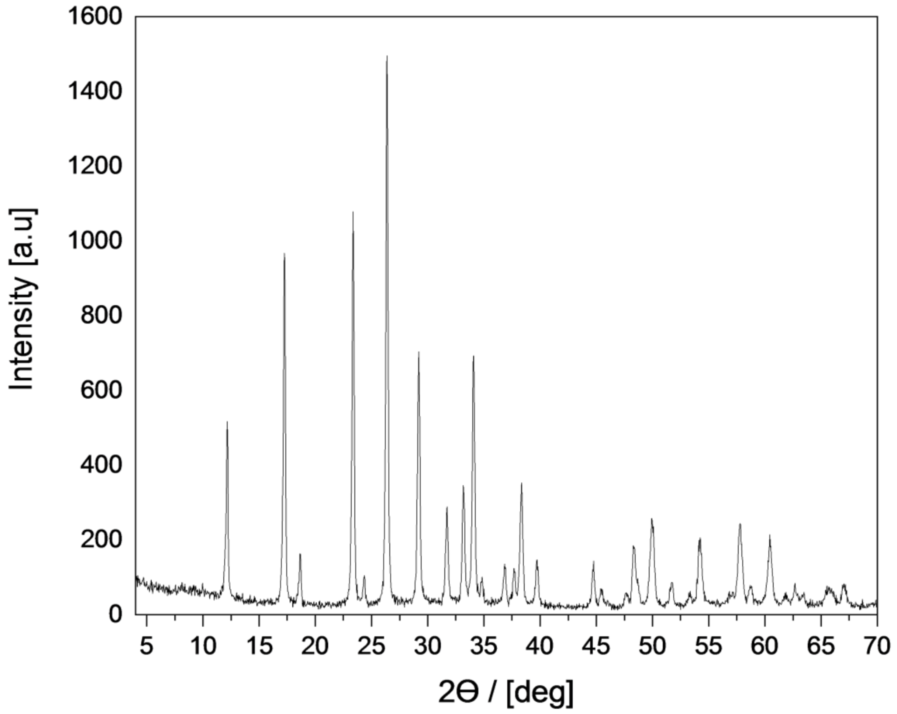

Figure S3. XRD of the decomposition product of compound **1**@2H_2O in air (after 5 days) ($NH_4Zn(OH)MoO_4$)

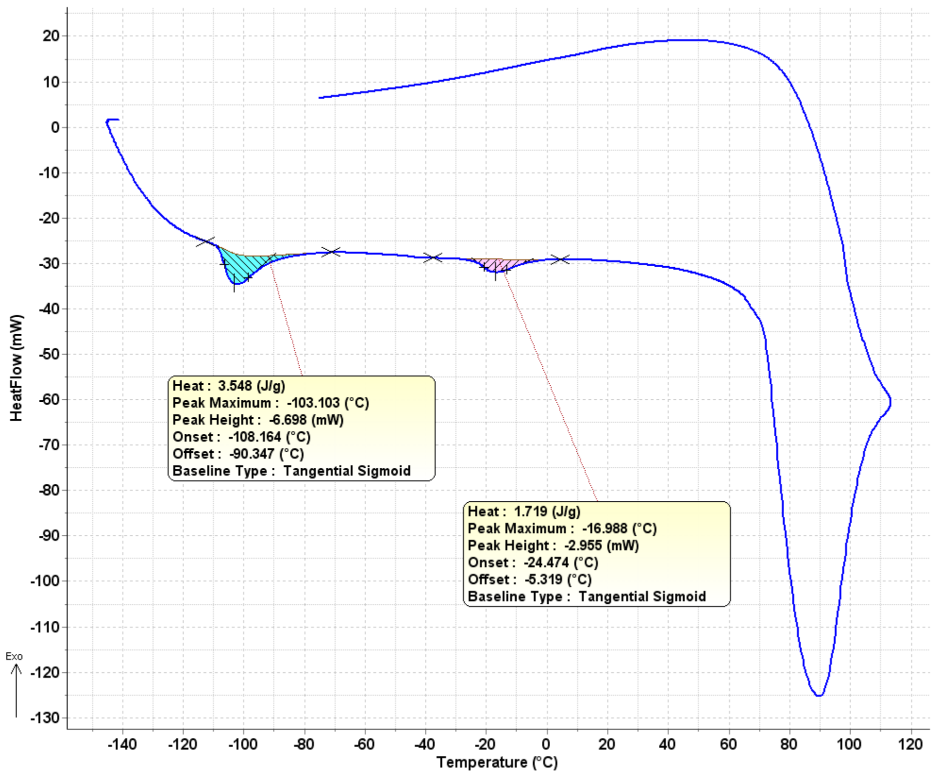

Figure S4. DSC results of compound 1@2H2O between -150 and +120 °C

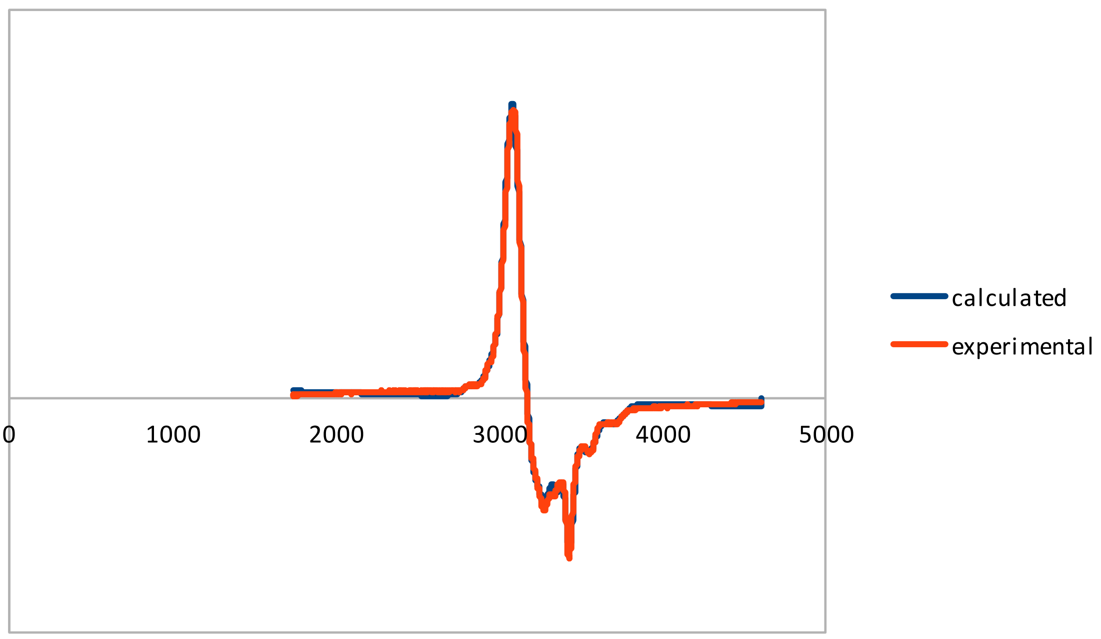

Figure S5. ESR spectra of powdered compound **2** at room temperature

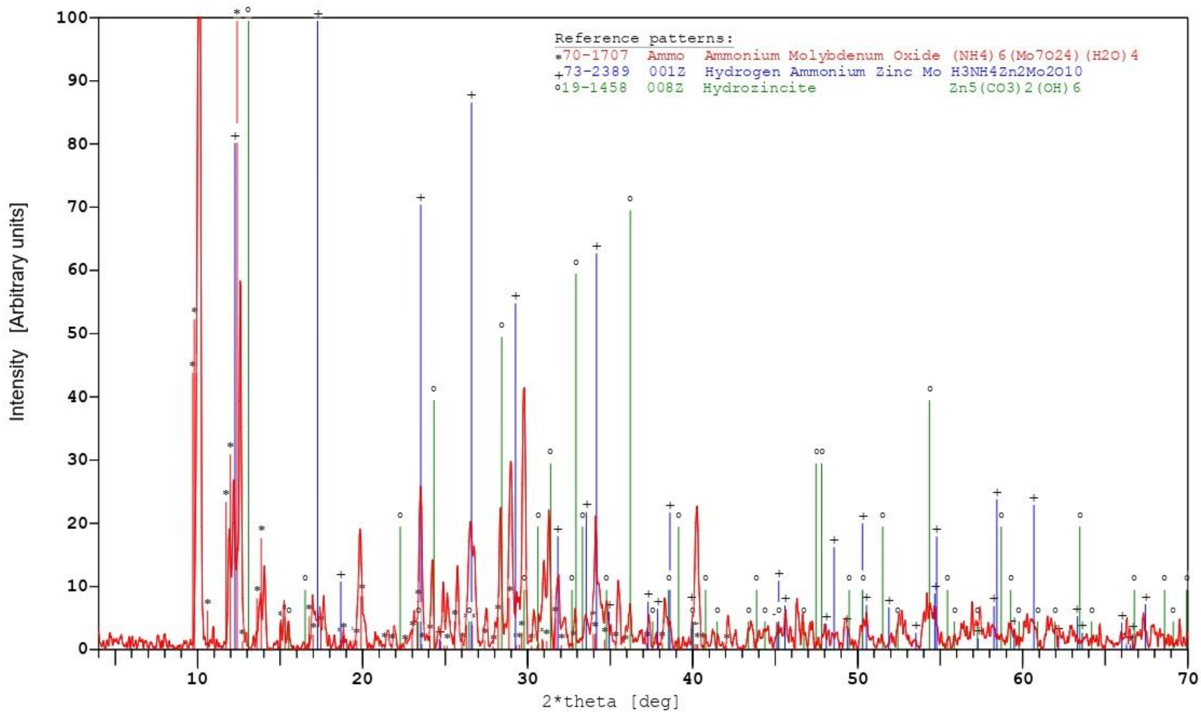

Figure S6. XRD of the hydrolysis product in aq. solution of compound **1**@2H_2O on boiling for 2 h

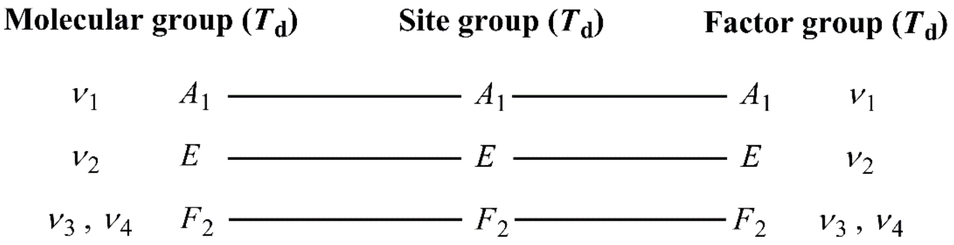

(a)

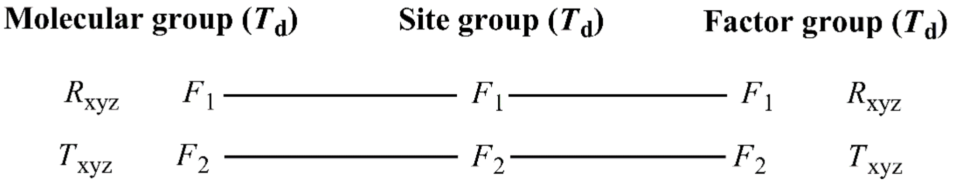

(b)

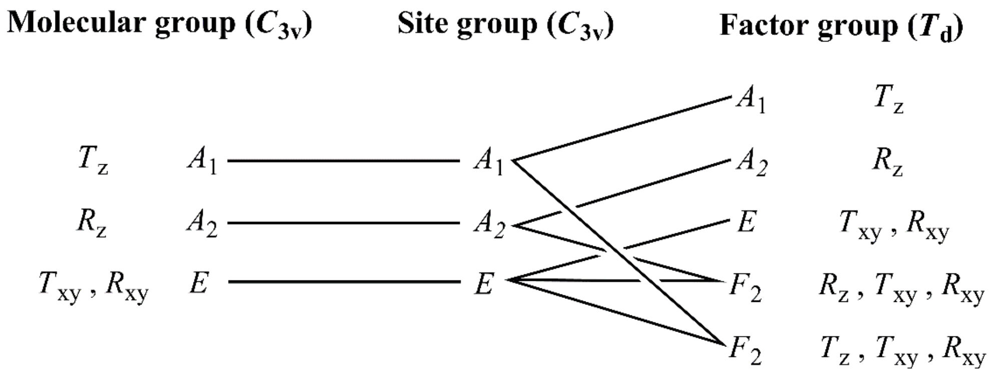

(c)

Figure S7. Correlation analysis of MoO_4^{2-} ion @2H_2O (a-internal modes, b-external modes) and ammonia external modes (c) in compound **1**

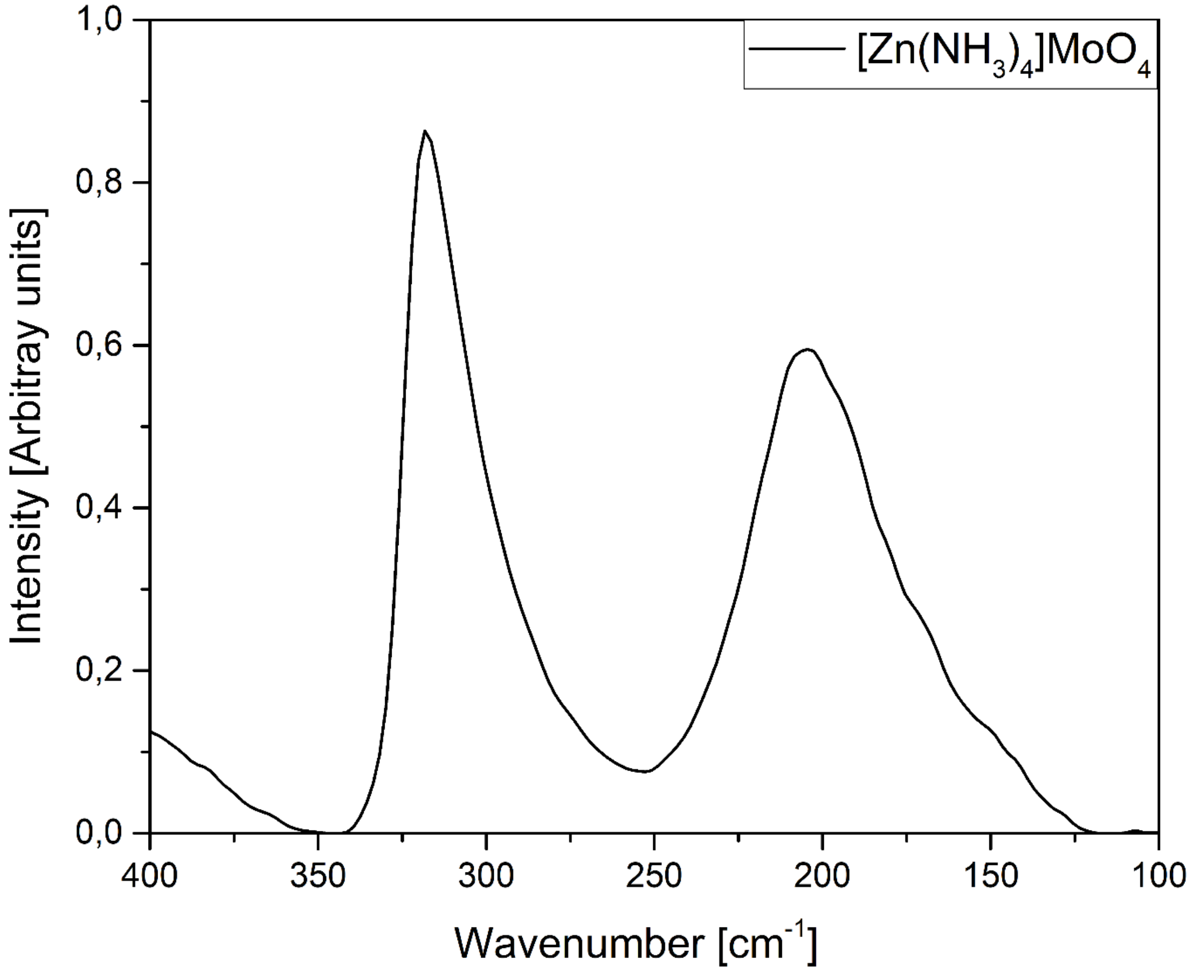

Figure S8. Far-IR spectrum of compound **1**@$2H_2O$

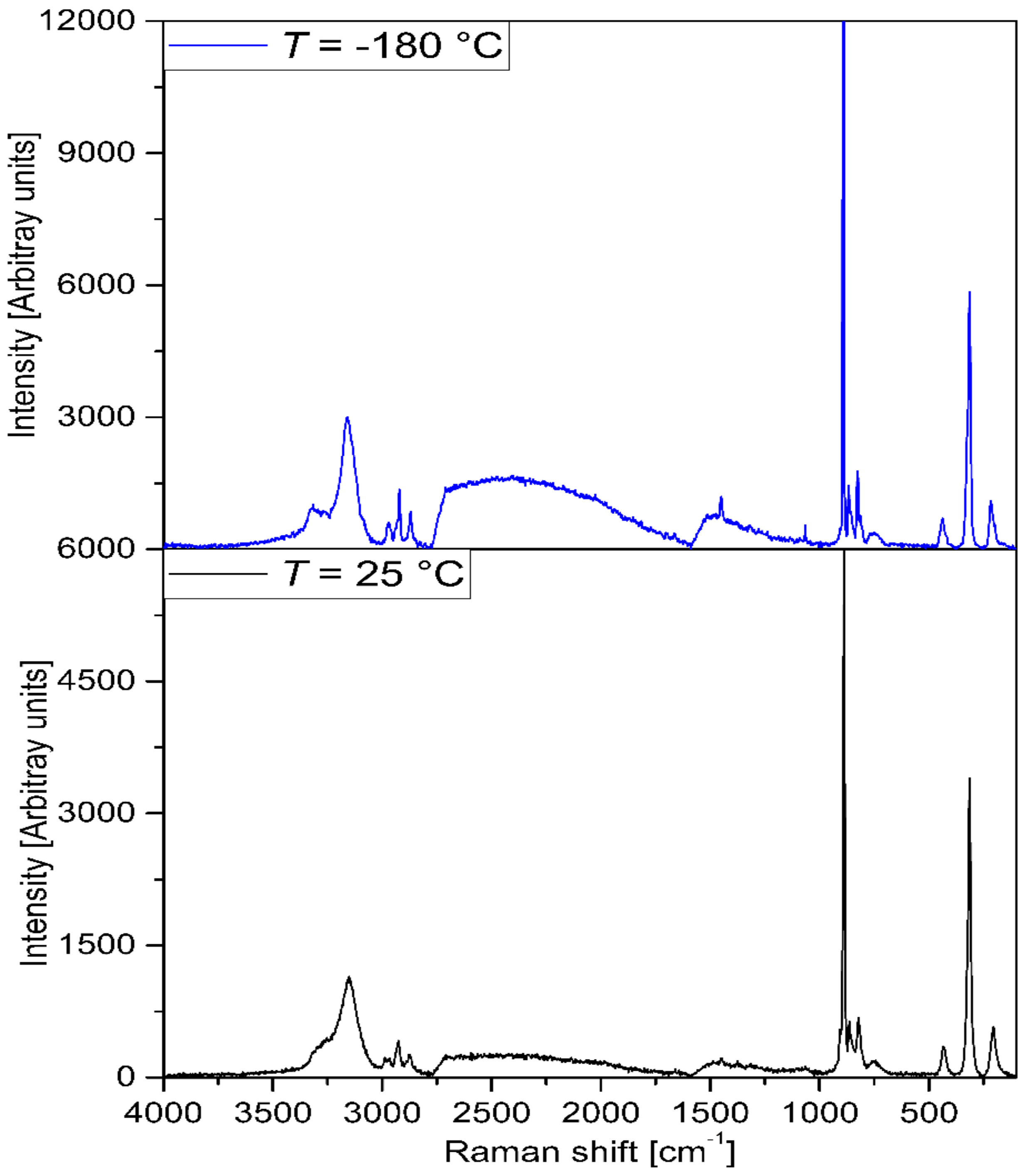

Figure S9. Raman spectra of compound **1** at -180 (a) and 25 °C (b) measured at 532 nm excitation

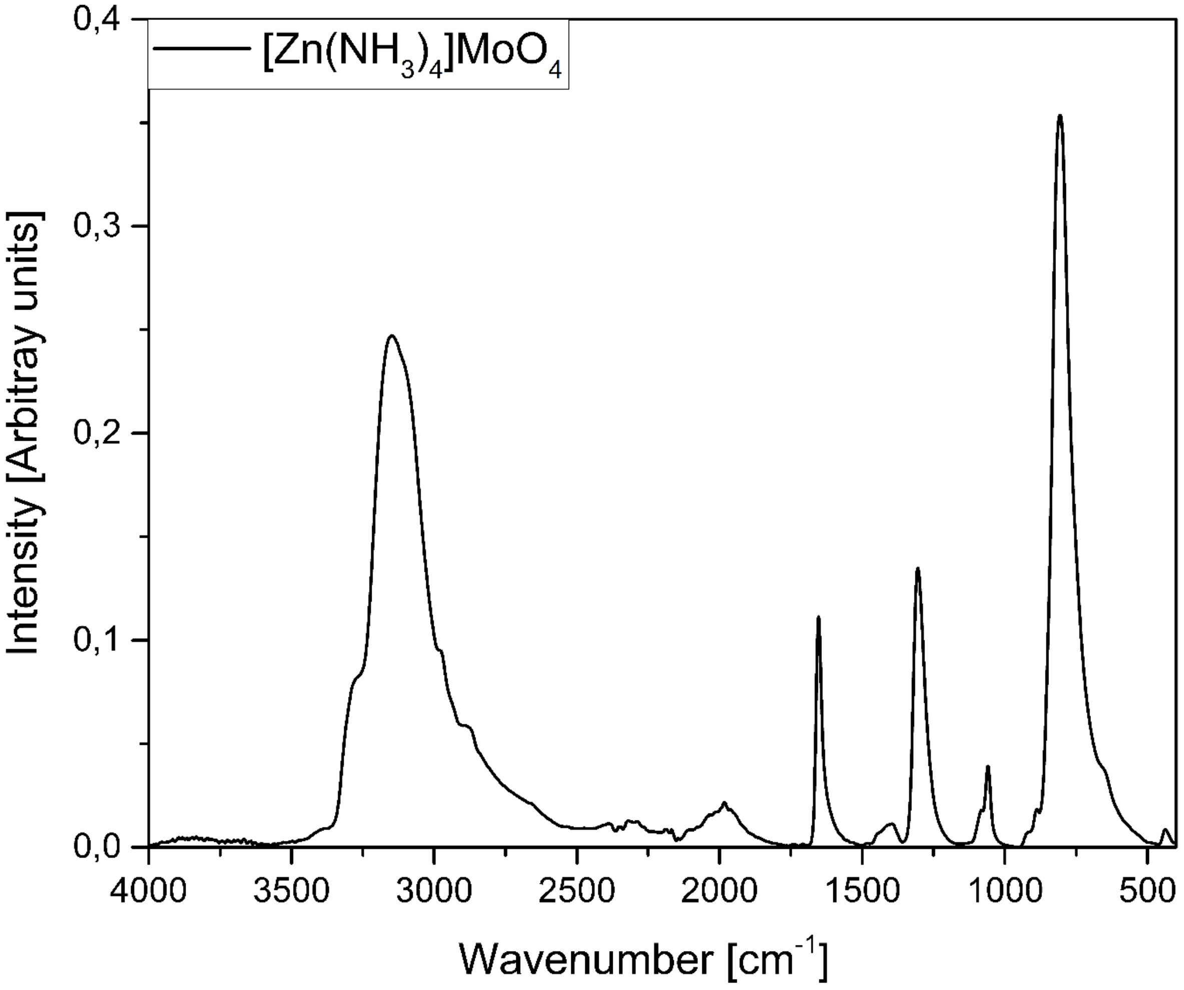

Figure S10. IR spectrum of compound **1**@$2H_2O$

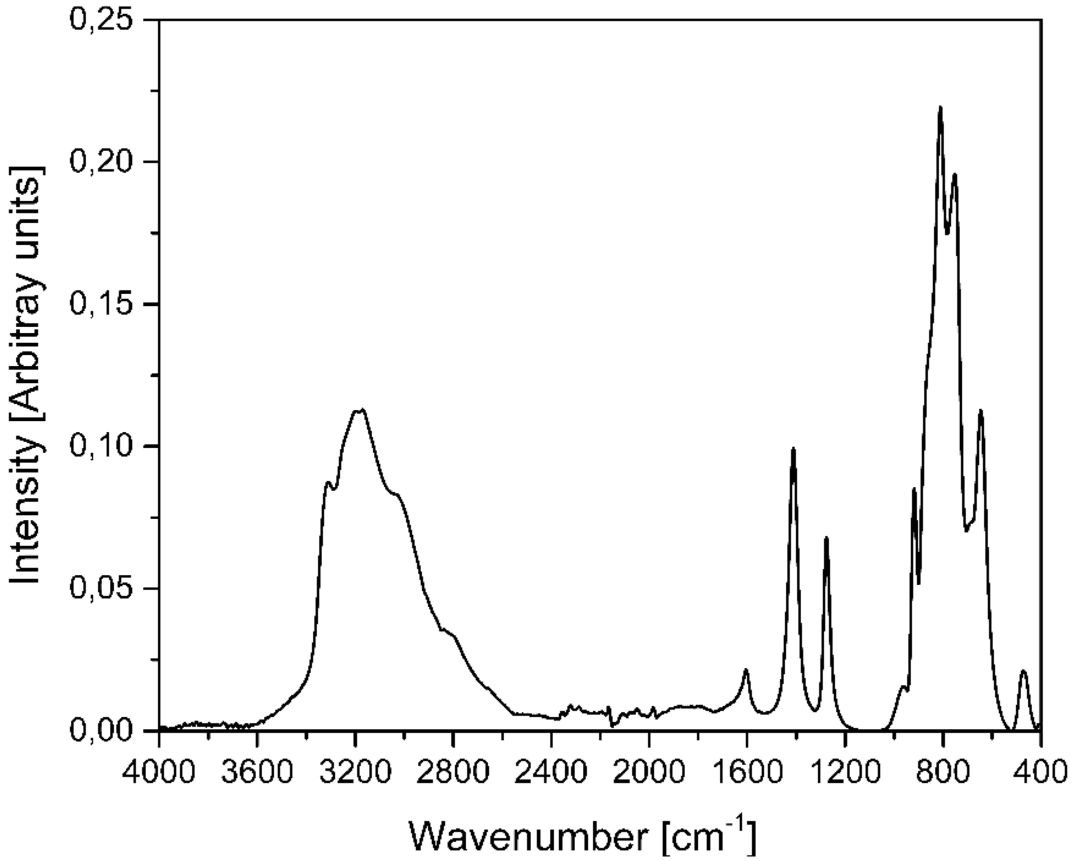

Figure S11. IR spectrum (ATR) of compound **2** at room temperature

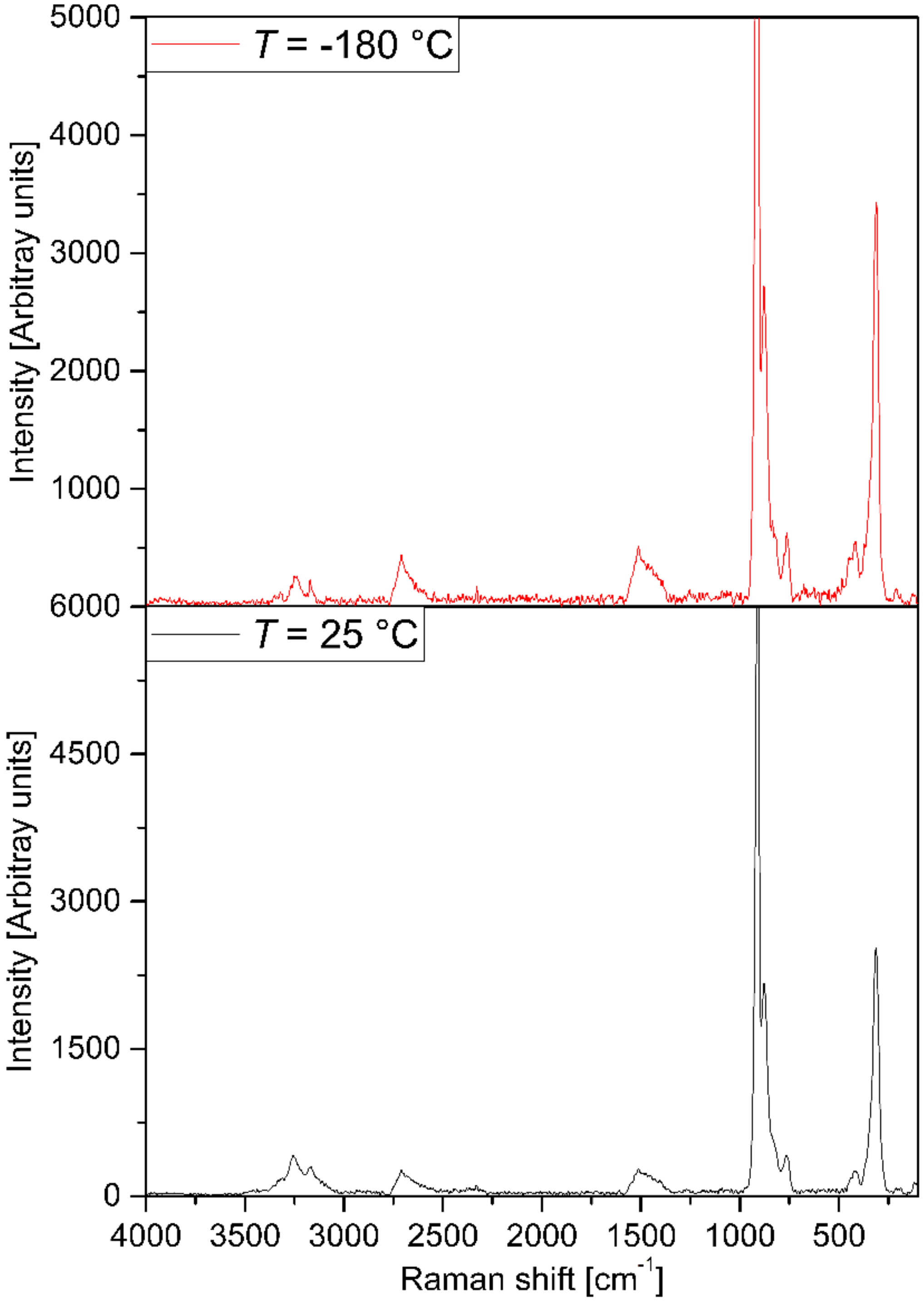

Figure S12. Raman spectrum of compound **2** at -180 (upper) and 25 °C (bottom) with 535 nm excitation

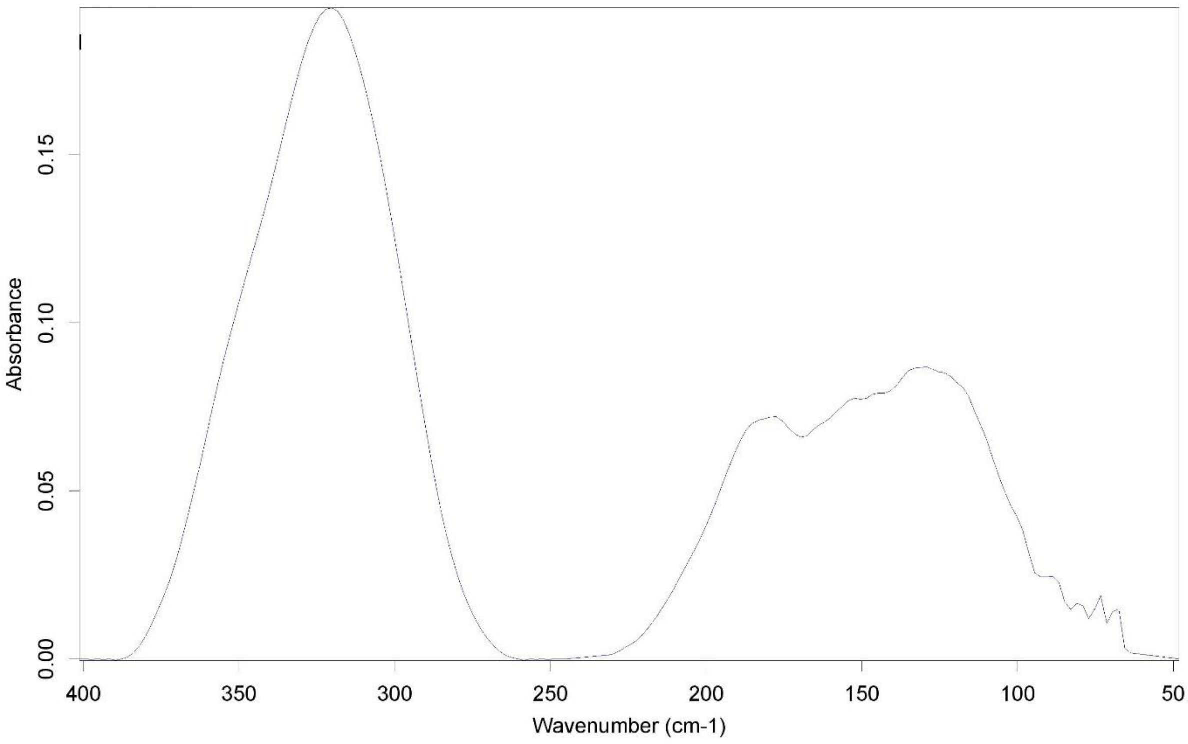

Figure S13. Far-IR spectrum of compound **2** at room temperature

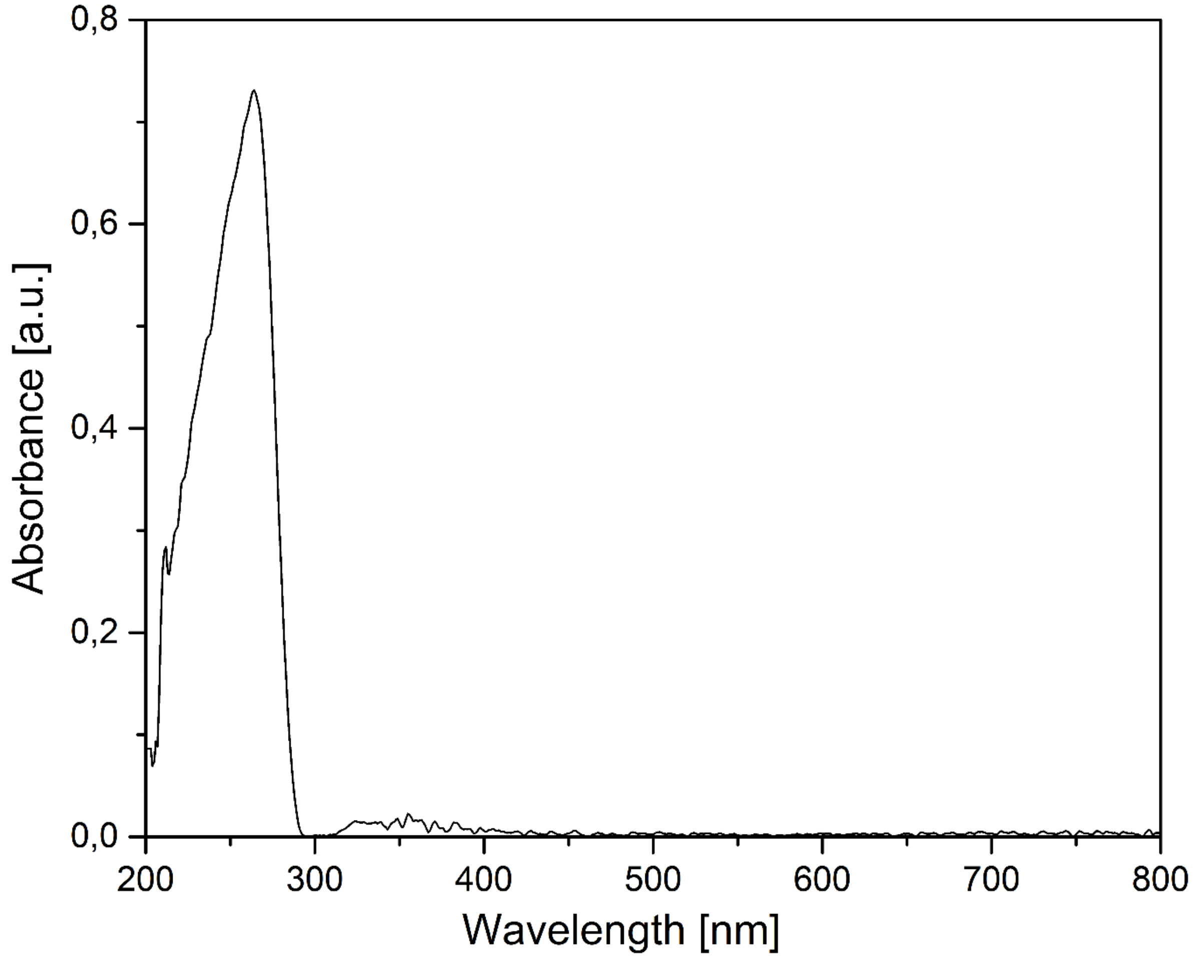

Figure S14. UV spectrum of solid compound **1**@2H_2O

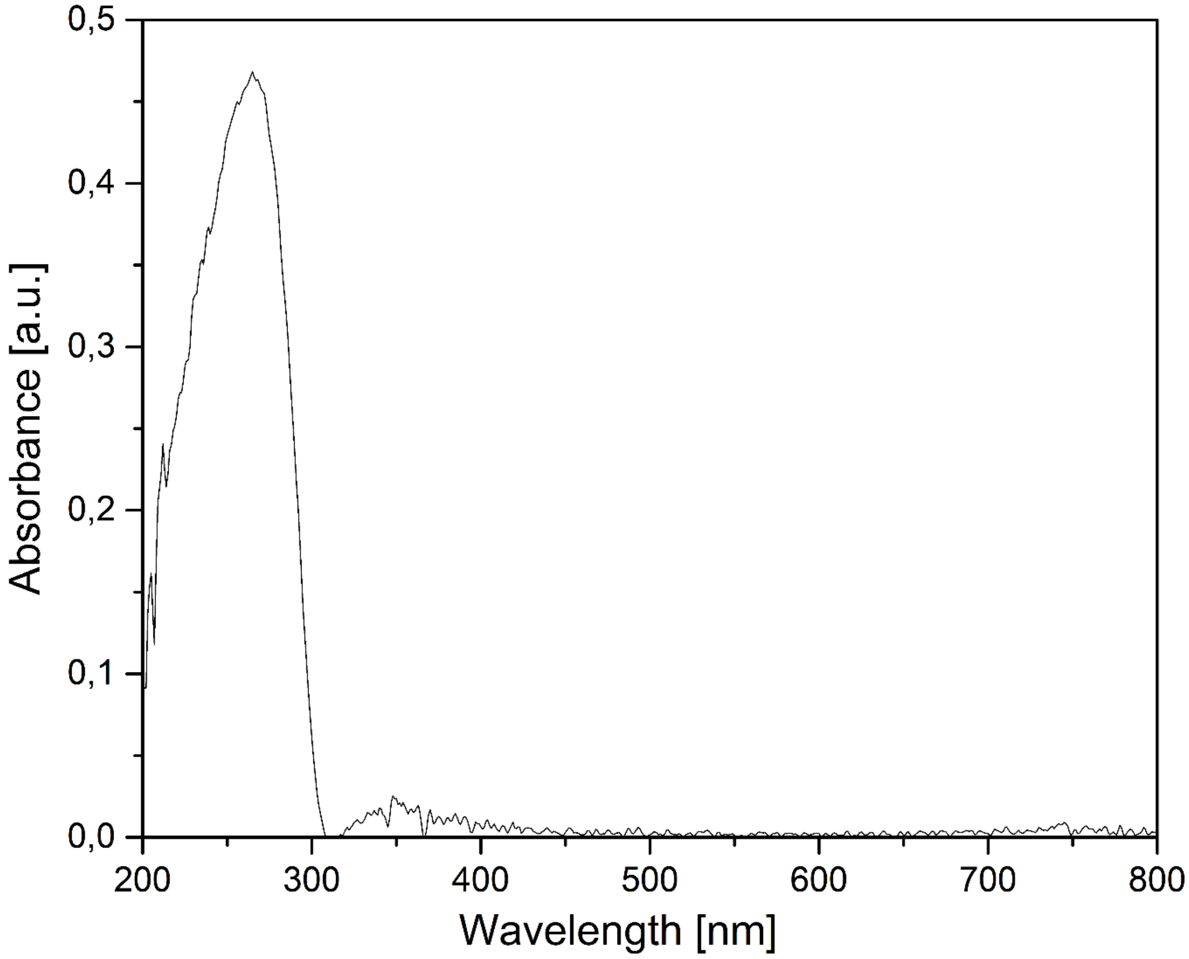

Figure S15. UV spectrum of solid compound **2**

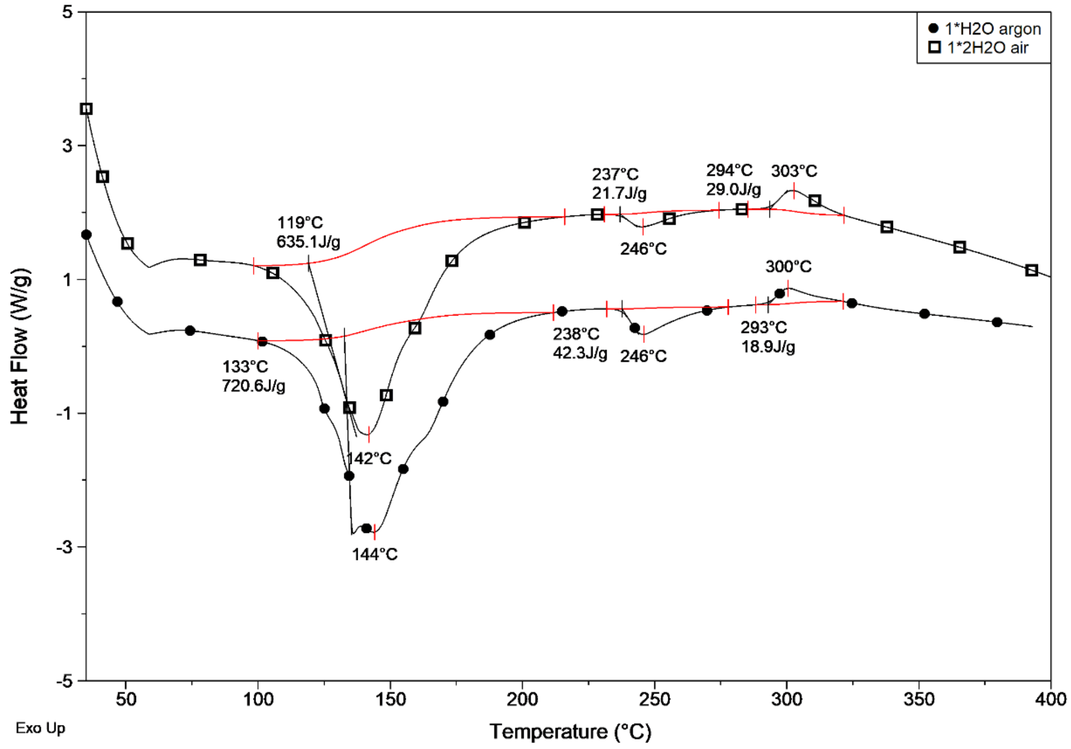

Figure S16. DSC of compounds **1** and **2** in air and argon atmosphere

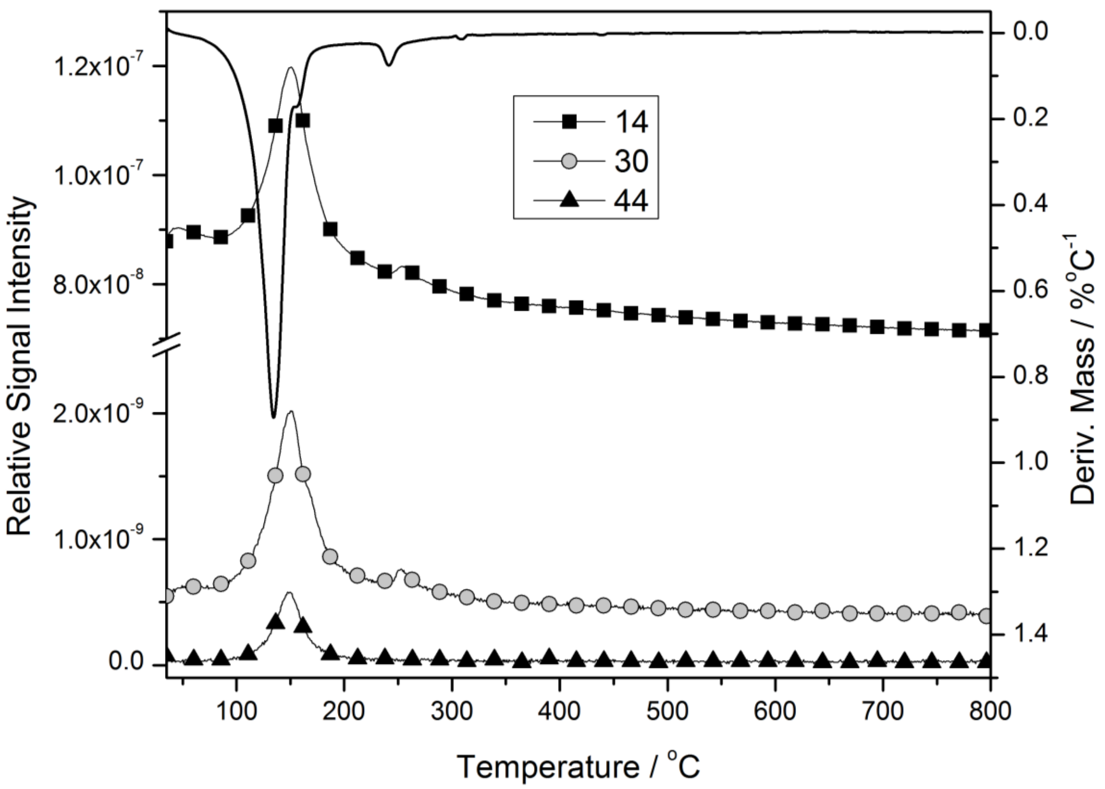

Figure S17 (a). TG-MS of compound **2** in air (m/z=14, 30 and 44).

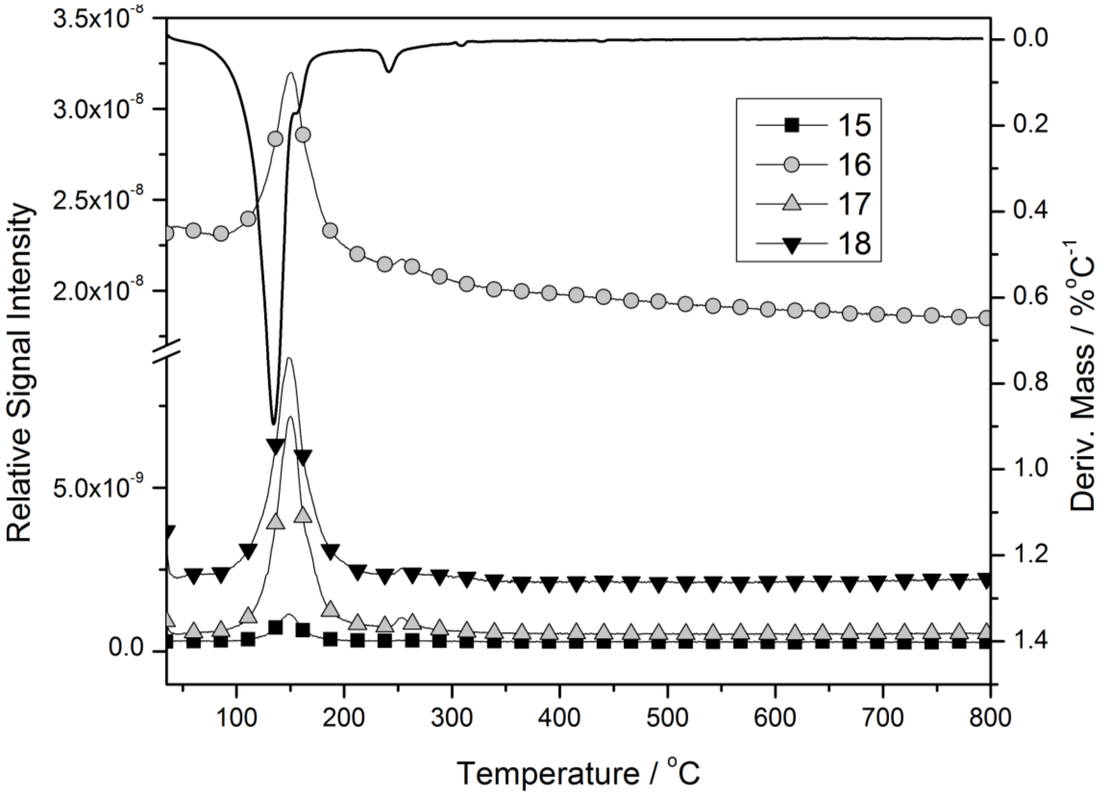

Figure S17 (b). TG-MS of compound **2** in air (m/z=15, 16, 17 and 18).

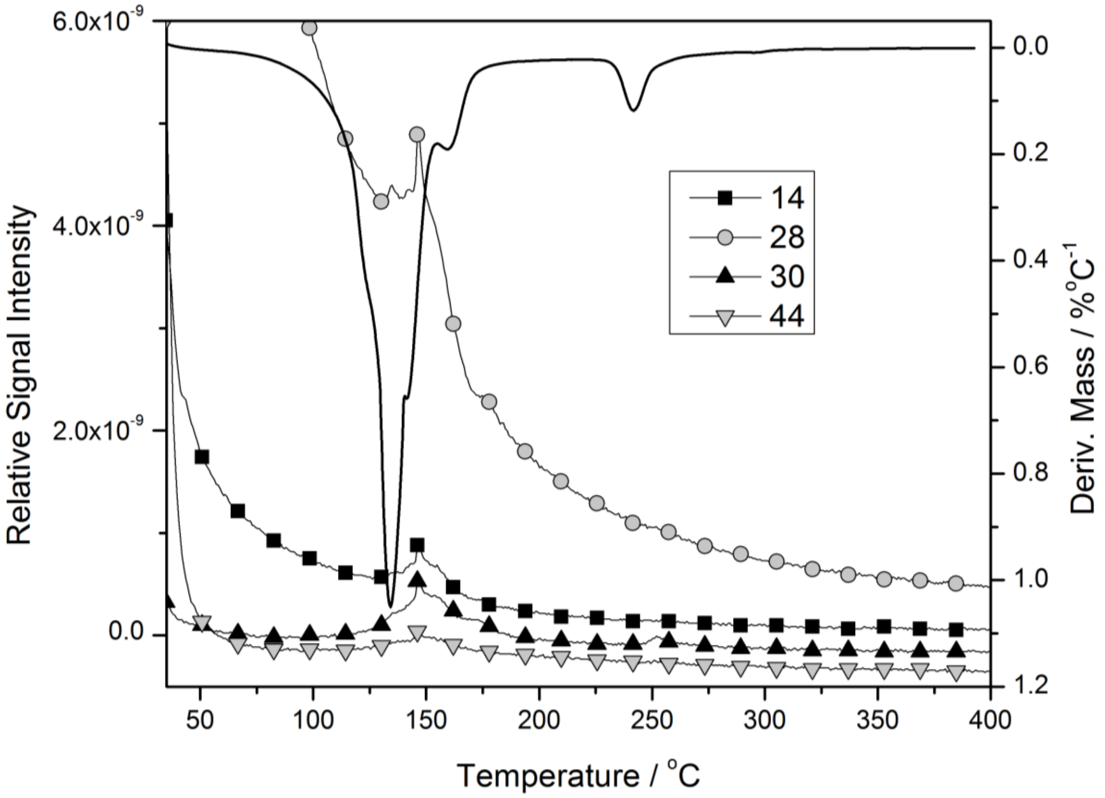

Figure S18 (a). TG-MS of compound **2** in argon (m/z=14, 28, 30 and 44).

.

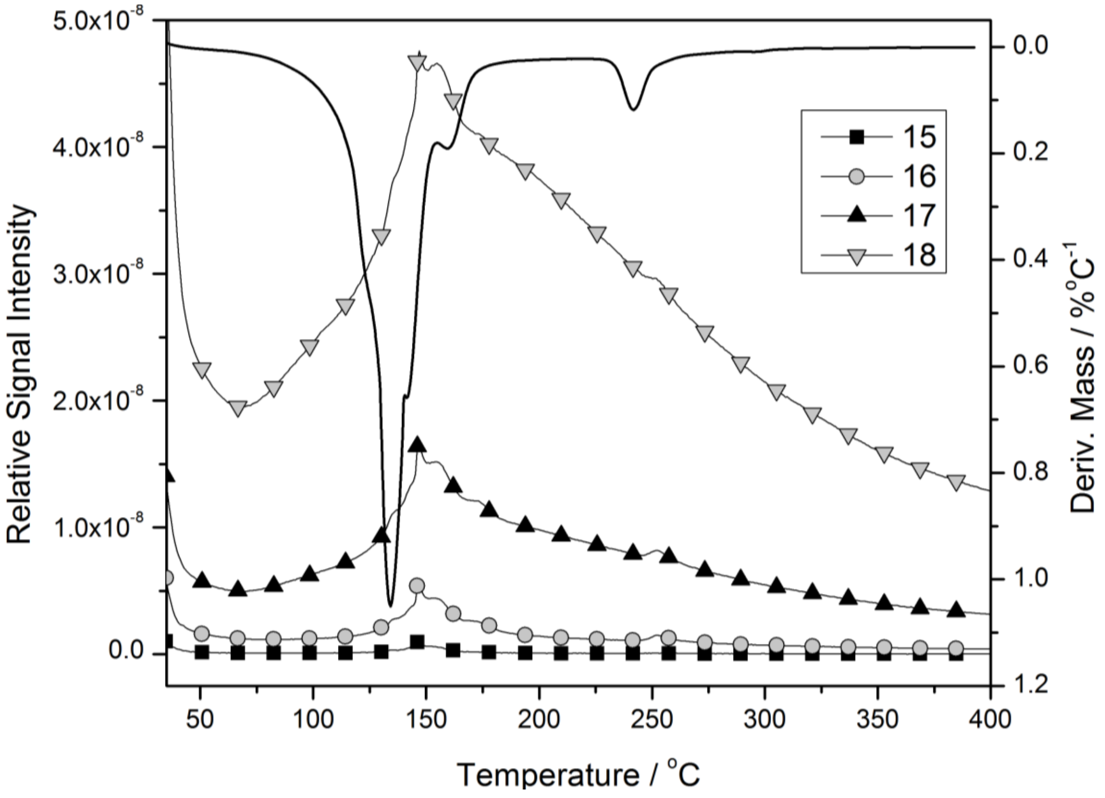

Figure S18 (b). TG-MS of compound **2** in argon (m/z=15, 16, 17 and 18).

Table S1. Miller indices of compound **1**@2H_2O.

Cubic, sg= F23
Unindexed=0, vol=1119.89 GOF=74.91 zero=0
a= 10.3846

**h**	**k**	**l**	**dc**	**do**	**do-dc**	**2Thc**	**2Tho**	**2Tho-2Thc**
1	1	1	5.9956	5.9883	-0.0072	14.7633	14.7813	0.0179
0	0	2	5.1923	5.1953	0.0030	17.0631	17.0530	-0.0101
0	2	2	3.6715	3.6656	-0.0059	24.2217	24.2613	0.0395
3	1	1	3.1311	3.1314	0.0003	28.4839	28.4811	-0.0028
2	2	2	2.9978	2.9964	-0.0014	29.7791	29.7929	0.0138
0	0	4	2.5961	2.5877	-0.0084	34.5199	34.6357	0.1158
3	3	1	2.3824	2.3819	-0.0005	37.7288	37.7369	0.0081
0	4	2	2.3221	2.3218	-0.0003	38.7476	38.7528	0.0052
4	2	2	2.1197	2.1172	-0.0026	42.6172	42.6711	0.0539
3	3	3	1.9985	1.9973	-0.0012	45.3415	45.3705	0.0290
0	4	4	1.8358	1.8352	-0.0005	49.6197	49.6354	0.0157
5	3	1	1.7553			52.0594		
0	0	6	1.7308	1.7307	-0.0000	52.8546	52.8559	0.0013
6	0	2	1.6419	1.6421	0.0001	55.9565	55.9519	-0.0046
5	3	3	1.5836			58.2099		
6	2	2	1.5655	1.5659	0.0004	58.9488	58.9340	-0.0148
4	4	4	1.4989			61.8497		
5	5	1	1.4541			63.9743		
6	0	4	1.4401	1.4402	0.0001	64.6739	64.6696	-0.0043
6	4	2	1.3877	1.3877	0.0000	67.4338	67.4314	-0.0023

Table S2: Zn−O and Zn−N bond distances of hexa- and pentacoordinated hydrated ammonia complexes formed by water addition to $[Zn(NH_3)_4]^{2+}$ calculated by density functional theoretical methods at the M05-2X/LANL2DZ level

Central geometry	Ligand arrangement	Bond length relationships	Zn-X distances, Å
CuN_4O_2 isomers			
OC-6	$CuN_4O_{1ax}O_{1eq}$	N_{eq}>O_{ax}	**Zn-N_{eq}=2.213**; Zn-O_{ax}=2.186;
OC-6	$CuN_{4O1ax}O_{2ax}$	O_{1ax}>N_{1ax};N_{4eq}	**Zn-O_{1ax}=2.231**; Zn-O_{2eq}=2.193; Zn-N_{1ax}=2.208; Zn-N_{2eq}=2.204; Zn-N_{3eq}=2.203; ZnN_{4eq}=2.198;
CuN_4O-isomers			
TB-5	ZnN_3-$O_{ax}N_{ax}$	O_{ax}>N_{ax}	**Zn-O_{ax}=2.269;** Zn-N_{ax}=2.220; ZnN_{eq1}=2.129; Zn-$N_{eq2,3}$=2.124;
TB-5	ZnN_2O-$N_{ax1,ax2}$	N_{ax}>O_{eq}<br>N_{eq}>O_{eq}	**Zn-$N_{ax1,2}$=2.212;** Zn-$N_{eq1,2}$=2.147; Zn-O_{eq}=2.106
CuN_3O_2 isomers			
TB-5	ZnN_3-O_{ax1}-N_{ax1}	O_{ax}>N_{eq}	**Zn-O_{ax1}=2.192;** Zn-O_{ax2}=2.185; Zn-N_{eq1}=2.111; Zn-N_{eq2}=2.108; Zn-N_{eq3}=2.103;
TB-5	ZnN_2O-N_{ax1},O_{ax1}	**O_{ax}>N_{ax}**	**Zn-O_{ax}=2.222;** Zn-N_{ax}=2.179; Zn-N_{eq1}=2.119; Zn-N_{eq2}=2.118; Zn-O_{eq1}=2.056;
SP-5	ZnN_2O_2-N_{ax}	**N_{eq}>$O_{eq1,2}$**	**Zn-$N_{eq1,2}$=2.164;** Zn-$O_{eq1,2}$=2.132; Zn-N_{ax}=2.108;
CuN_2O_3 isomers			
TB-5	ZnN_2O-O_{ax1},Oa_{x2}	O_{ax1}≈O_{ax2}>$N_{eq1,2}$	**Zn-O_{ax1}=2.153;** Zn-O_{ax2}=2.152; Zn-$N_{eq1,eq2}$=2.101; Zn-O_{eq}=2.033;
TB-5	$ZnNO_2$-O_{1ax},N_{1ax}	N_{ax1}>O_{ax1}	**Zn-N_{ax1}=2.144;** Zn-O_{ax1}=2.137; Zn-N_{eq1}=2.109; Zn-$O_{eq2,3}$=2.058;
TB-5	ZnO_3-$N_{ax1,ax2}$		**Zn-$N_{ax1,2}$=2.129;** Zn-$O_{eq1,2,3}$=2.101;
$ZnNO_4$ isomers			
SP-5	ZnO_3N-O_{ax}	O_{ax}≈N_{eq}	**Zn-O_{ax}=2.088; Zn-N_{eq}=2.087;** Zn-$O_{eq1,2}$=2.065; Zn-O_{eq3}=2.087;
TB-5	ZnO_4-N_{eq}	$O_{ax1,2}$>N_{eq}	**Zn-$O_{ax1,2}$=2.109 (2x);** Zn-$O_{eq1,2}$=2.064 (2x); Zn-N_{eq}=2.090

TB-5	ZnO_4-N_{ax}	N_{ax}>O_{eq}	**Zn-N_{ax}=2.121;** Zn-O_{eq1}=2.103; Zn-O_{eq2}=2.081; Zn-O_{eq3}=2.046; Zn-O_{eq4}=2.045;

Table S3: Assignment of the anion vibrational modes in compound **1@**$2H_2O$

**Assignment**	**Raman**		**IR**
	25 °C	-180 °C	25 °C
ν_s (Mo-O) (A_1)			
δ_s (Mo-O) (E)	316vs|	316vs|	315vw|
ν_{as} (Mo-O) (F_2)	864w, 822w	864w, 822w	799vs
δ_{as} (Mo-O) (F_2)	|432w*	432w*	433w*|
*Mixed/overlapped mode with ν_{as}(Zn-N) (F_2); s-strong; vs-very strong; w-weak; vw-very weak;			

Table S4: Assignment of cation vibrational modes in compound **1**@2H_2O.

Assignment	Compound **1**@2H_2O			$Zn(NH_3)_4(MnO_4)_2$, Ref.12	
	Raman		IR	Raman	IR
	25 °C	-180 °C	25 °C	25 °C	25 °C
ν_s(Zn-N) (A_1)	-	-	450 w	-	451 w
δ_s(Zn-N) (E)	205	216	-	-	232w
ν_{as}(Zn-N)	433w*	437w*	433w*	426w	421w
δ_{as}(Zn-N)	205	216w	-	-	179 s
ν_s(N-H) (A_1)	3158	3148	3143 vs	3163	3253m
δ_s(N-H)(A_1)	-	-	1263m	-	1219s
ν_{as}(N-H) (E)	3319	-	3276s	3326	3329vs
δ_{as}(N-H) (E)	-	-	1652m,** 1621sh	-	1612m
$\rho(NH_3)$ (E)	-	-	~700sh, ~650sh	695	716w, 690w
*Mixed mode with δ_{as}(Mo-O); **Mixed mode with δ_{as}(N-H)					

Table S5: Comparison of IR and Raman data for $NH_4Zn(OH)MoO_4$ (compound **2**) and $NH_4Cu(OH)MoO_4$ (compound **2**-Cu).

Assignation	Compound **2**			Compound **2**-Cu	
	Raman		IR	Raman	IR
	25 °C	-180 °C	25 °C	25 °C	25 °C
ν_s(Mo-O)(A_1)	910 vs	910 vs	918m	905	921
δ_s(Mo-O)(E)	316 m	319 m	316 m	-	320
ν_{as}(Mo-O)(F_2)	875 m	877 m	867sh, 813vs, 751vs	868, 825, 750	884, 819, 761
δ_{as}(Mo-O)(F_2)	411 w	441,414 w	400w	-	-
ν_s(N-H) (A_1)	-	-	3030s	-	3012
δ_s(N-H)(E_1)	-	-	1605m	-	1602
ν_{as}(N-H) (F_2)	-	-	3199s, 3168s	-	3235
δ_{as}(N-H) (F_2)	-	-	1410m	-	1413
δ(Zn/Cu-OH)	-	-	650w	-	696
ν(Zn/Cu-O-Mo)	-	-		-	618
Zn/Cu-O-Zn/Cu bridge	-	-	1278m	-	1288
ρ(H_2O)	-	-	960m	-	-
$2\delta_{as}$(N-H)	-	-	2805w	-	2807
ν_s(NH)+ν_L(lattice), ν(OH)	-	-	3314s	-	3414, 3478

Table S6: Apparent Reaction Rate Constants of the Photodegradation of Methyl orange and Congo Red at 375 nm Irradiation (18 W) in the presence of 0.03% catalysts

Catalyst	Substrate	pH	$k_{apparent}$ [10^{-4} min^{-1}]	R^2
Without catalyst	Methyl Orange	5.6	1.0	0.92
Compound **1**@2H_2O	Methyl Orange	5.6	2.0	0.87
Compound **2**	Methyl Orange	5.6	2.0	0.97
Compound **10**-220-**1**	Methyl Orange	5.6	0.7	0.98
Compound **10**-350-**1**	Methyl Orange	5.6	2.0	0.88
Compound 10-350-**2**	Methyl Orange	5.6	2.0	0.96
Without catalyst	Congo Red	5.7	1.0	0.98
Compound **1**@2H_2O	Congo Red	5.7	11	0.99
Compound **2**	Comgo Red	5.7	26	0.99
Compound **10**-220	Congo Red	5.7	38	0.99
Compound **10**-350-**1**	Congo Red	5.7	9	0.99
Compound **10**-350-**2**	Congo Red	5.7	3	0.96
